# A vegetable-induced hemolytic crisis in a G6PD deficient person: a case report

**DOI:** 10.1186/s13104-018-3286-9

**Published:** 2018-03-14

**Authors:** N. D. B. Ehelepola, A. N. Abayagunawardana, T. N. Sudusinghe

**Affiliations:** Teaching (General) Hospital–Kandy, Kandy, Sri Lanka

**Keywords:** G6PD deficiency, Hemolytic anemia, Glucose-6-phosphate dehydrogenase deficiencies, *Acalypha indica*, Leptospirosis, Case report, Sri Lanka

## Abstract

**Background:**

Hemolysis can occur in people with G6PD deficiency under oxidative stress. *Acalypha indica* is a tropical plant used as a medicinal plant as well as a vegetable. There are a few reported cases of Acalypha *indica* ingestion induced hemolysis in G6PD deficient people. All except one of them are from Sri Lanka. The information available at present (2017) about G6PD deficiency prevalence and variants of the G6PD gene among Sri Lankans is very sparse. There are no past reports on hemolytic crisis in a G6PD deficient person presenting mimicking leptospirosis.

**Case presentation:**

A middle-aged Sri Lankan man presented on the third day of illness complaining of fever, head ache, arthralgia, myalgia, abdominal pain, vomiting, passing dark urine and reduced of urine volume. He gave a history of possible exposure to leptospirosis. He was pale, icteric and his liver was palpable 1 cm below costal margin and there were no other remarkable findings upon physical examination. He had neutrophilic leucocytosis. Leptospirosis was diagnosed. During the second assessment we noticed he was very pale and his urine sample pointed towards hemoglobinuria. Further questioning revealed he had consumed leaves of Acalypha *indica* as a vegetable. Acute hemolysis in a G6PD deficient patient following *Acalypha indica* ingestion was diagnosed. Blood transfusions were given to correct his anemia. Later, Brewer’s test and quantitative assay of G6PD levels confirmed the diagnosis of G6PD deficiency.

**Conclusions:**

A hemolytic crisis following oxidative stresses in G6PD deficient patients can present mimicking leptospirosis. Further investigations may reveal why the great majority of cases of acute hemolysis in G6PD deficient person following *Acalypha indica* ingestion are from Sri Lanka.

## Background

Glucose-6-Phosphate Dehydrogenase (G6PD) is the enzyme that protects red blood cells against oxidative stresses [1–3]. G6PD deficiency is caused by point mutations in the coding region of the G6PD gene in the X chromosome [2–4]. The disease has a broad spectrum of biochemical and clinical phenotypes [1, 3]. G6PD deficient people are usually asymptomatic until exposure to an oxidative stress in the form of a drug such as primaquine, a food such as fava bean, a cosmetic such as henna (hena), a household chemical such as naphthalene or an infection that can result in acute hemolytic anemia [1–4]. The foundation of the management of G6PD deficiency is avoiding oxidative stresses [2]. Blood transfusions and iron and folic acid supplements are administered after an episode of acute hemolysis [2]. It is the commonest enzyme deficiency worldwide affecting 400 million people [2, 3]. It is more prevalent in regions where malaria was endemic in Sri Lanka as well as in malaria endemic regions of tropical Africa, the Middle East, Mediterranean and South East Asia and provides some protection against malaria [1–4].

The information available at present (2017) about G6PD deficiency prevalence and variants of G6PD gene among Sri Lankans are very sparse [3, 4]. The overall prevalence of G6PD deficiency in Sri Lanka is less than three percent of the population [3]. Nevertheless, in some ancient villages where malaria has been endemic for centuries the frequency of occurrence is as high as 20.9% [3]. A recent Sri Lankan study demonstrates that males and females are equally affected by G6PD deficiency [3]. The only detailed report on G6PD gene variants among Sri Lankans is a study done in the Kataragama area [4]. Common G6PD gene variants reported among other Asian populations were rare or absent and 17 genetic variants were polymorphic in that population and the mutant allele was the major allele in 9 SNPs (single-nucleotide polymorphisms). [4]. Nine out of the 17 SNPs detected had a minor allele frequency greater than 10% and those seemed to be providing some protection against *Plasmodium falciparum* in males [4].

**Fig. 1:**
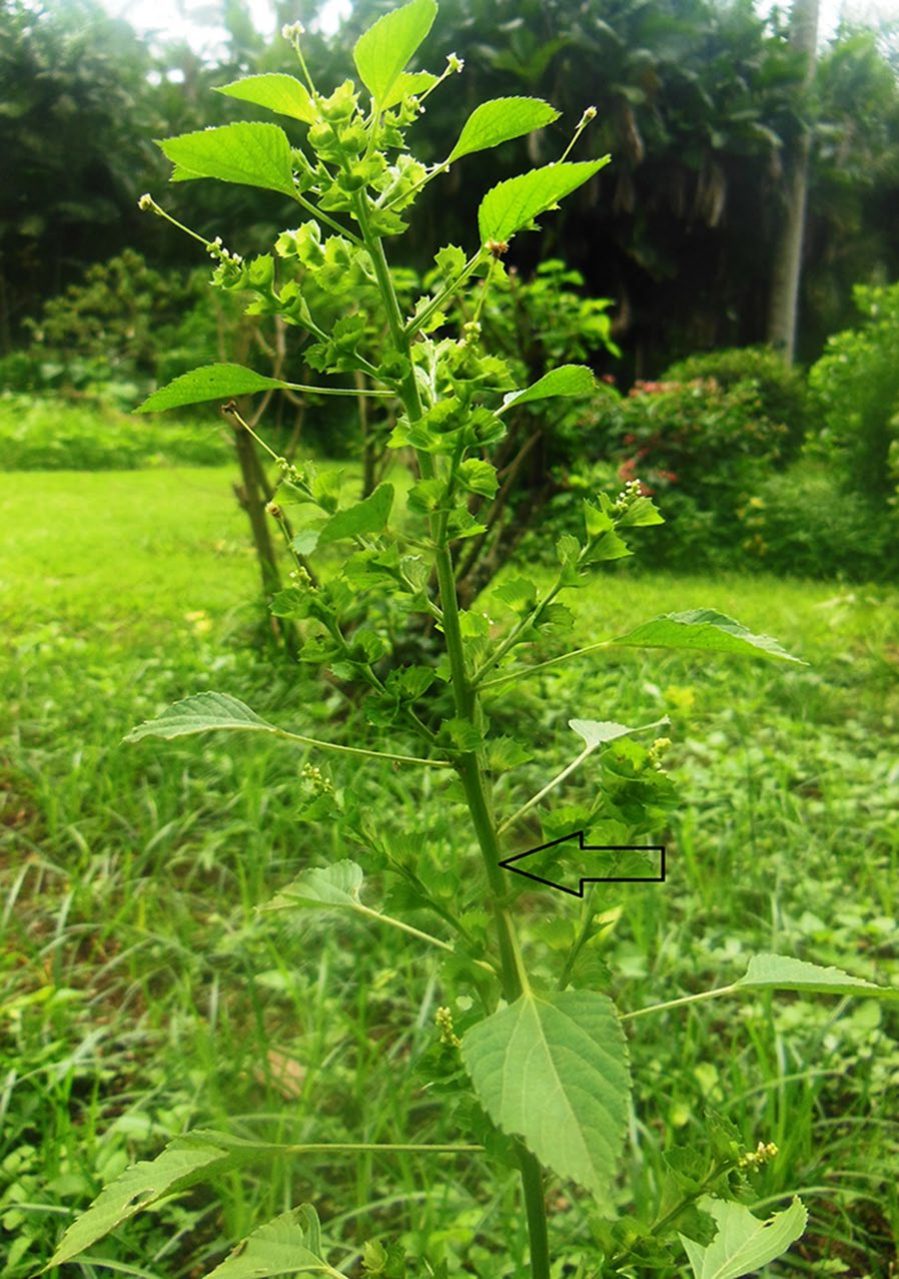
An *Acalypha indica* plant (arrow head), a small one, locally known as Kuppameniya grown at the Royal botanical gardens, Peradeniya, Sri Lanka

*Acalypha indica,* locally known as Kuppameniya (Fig. 1) is a tropical plant used in South and South East Asia, Africa and the Americas as a medicinal plant in local alternative medical practices as well as a vegetable [5].

There are few reported cases of the consumption of it as an alternative medicine, causing hemolysis in G6PD deficient patients and all but one of them are from Sri Lanka [6–9]. An acute hemolytic crisis in a G6PD deficient patient can give rise to symptoms and signs [10] that mimic a leptospirosis infection [11] as we describe below, making initial clinical diagnosis more challenging and we did not find any similar cases reported before.

## Case presentation

A 45 year old Sri Lankan man presented to us with a 2 day history of fever, head ache, arthralgia, myalgia especially affecting the lower limbs and lower abdominal pain. In addition he had vomiting and passing of dark colored urine for one day. He said that there was an apparent reduction of volume of urine he passed. The day before he had taken treatment from a general practitioner and has been on oral amoxicillin, paracetamol (acetaminophen) and vitamin B complex tablets since then. As his symptoms got aggravated he was admitted to our hospital.

He is used to standing bare footed at the mouth of a large urban drain (polluted with animal excreta) discharging into a stream after the rains and catching fish because he noticed the fish of the stream concentrating there to eat matter flushed along the drain. The last time he did this was 6 days before admission. That indicates his exposure to leptospirosis. His past medical history and the family history were unremarkable except that there was a history of an allergic reaction to an unidentified agent 2 decades ago that needed hospitalization.

Upon examination there was a mild pallor and icterus but no conjunctival suffusion, no fever. His pulse rate was 88/min, blood pressure was 120/80 mm mercury, his lungs were clear to auscultation and his liver was felt 1 cm below the right costal margin. The results of his central nervous system examination were normal. There was neutrophilic leucocytosis. Leptospirosis was diagnosed and intravenous ceftriaxone and supportive therapy was initiated.

**Fig. 2:**
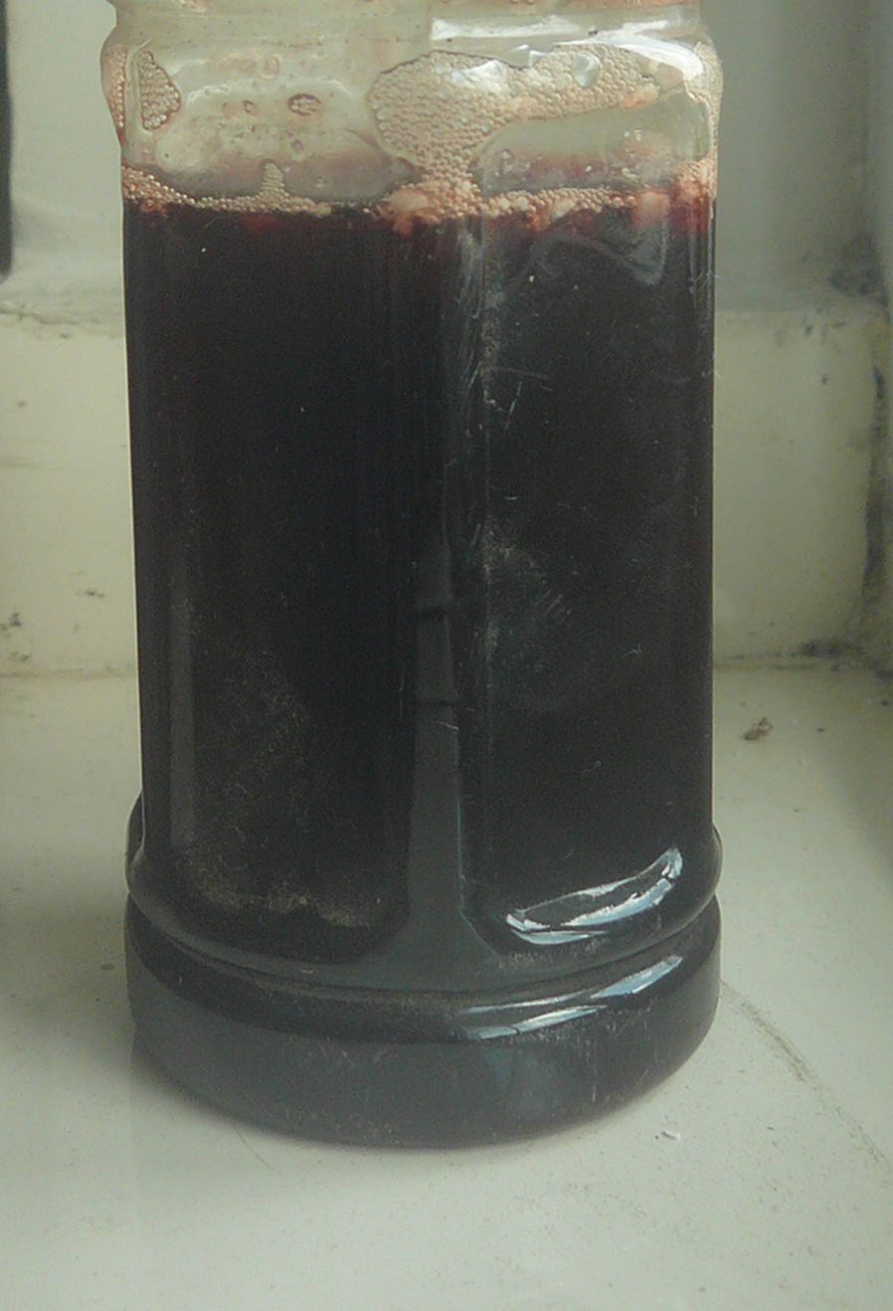
A urine sample from the patient on third day of the illness

Upon the second assessment the patient was very pale and a sample of urine from him indicated hemoglobinuria as shown in Fig. 2.

Further details were asked and he revealed that he had eaten cooked leaves of Kuppameniya (*Acalypha indica)* 1 day before the onset of fever, but denied any diagnosed hematological disease affecting his brother or relatives from the maternal side of his family. He did not know about any illness during his neonatal period.

Hence we started a workup for the possibility of hemolytic anemia as well. Table 1 summarizes his laboratory investigation results.

**Table 1:** Synthesis of his laboratory investigation results (important ones are in italics fonts)

Test	Reference range	The day of the illness				
		Day 3	Day 4	Day 5	Day 6	Day 7
White cell count (× 10^9^/l)	***4.0–10.0***	***24.10 → 24.13***	***30.28***	***22.15***		***9.8***
Neutrophils %	***50–70***	***64.9 → 65.3***		***86.18***		***64***
Lymphocytes %	20–40	28.8 → 28.3		11.3		35
Hemoglobin (g/dl)	11–16	7.7 → 7.3	5.9	6.2		8.0
Hematocrit (%)	37–54	22.7 → 22.5	18.6	17.62		24.3
Platelets count (× 10^9^/l)	150–450	280 → 287	315	309		386
Serum lactate dehydrogenase (LDH) in U/l	***140–330***	***6840***				
Reticulocyte count %	***0.3–3.0***	***8.71***				
Serum alanine transaminase (ALT) in U/l	7.0–45	21.1	29			
Serum aspartate transaminase (AST) in U/l	13.0–31.0	82.0	114			
Serum alkaline phosphatase (ALP) in U/l	53.0–128.0	98				
Serum bilirubin—total (micro mol/l)	***3.0–21.0***	***115.1***				
Serum bilirubin—direct (micro mol/l)	1.0–7.0	11.9				
Serum gamma-glutamyltransferase (GGT) in U/l	15.0–30.0	17				
Blood urea (mmol/l)	2.10–7.10	7.09				
Serum creatinine (mg/dl)	0.90–1.30	0.58	0.6			
Serum sodium (mmol/l)	133–148	142				
Serum potassium (mmol/l)	3.5–5.5	5.0				
Chest X ray		Normal				
12 lead electrocardiogram		Normal				
Blood picture (blood taken day 2 and report arrived day 4)	***Normocytic normochromic red cells with marked polychromacia, red cell fragments and blister and bite cells were seen. Absolute neutrophil leucocytosis with left shift. Platelets were normal. Summary: Evidence of hemolysis compatible with that due to oxidant stress on a G6PD deficient patient***					

He denied ingestion of other drugs or food that may induce hemolysis. A hemolytic crisis triggered by *Acalypha indica* in *a* G6PD deficient person was diagnosed, antibiotic was stopped. Blood transfusions were given (700 ml packed cells during the day 3, 350 ml each on day 4 and day 6). A consultant hematologist’s opinion obtained. After his symptoms subsided on the fifth day after admission, he was sent home on ferrous sulfate 400 mg and ascorbic acid 100 mg thrice daily, folic acid 1 mg daily supplements with a list of drugs to be avoided. Reticular cell count was repeated 6 weeks after discharge. It has returned to normal (0.5%). Patient was again referred to the hematologist, a positive Brewer’s test confirmed the diagnosis. Brewer’s test [12] is the only test available at Sri Lankan state hospitals like ours for confirmation of G6PD deficiency [3]. Later a G6PD qualitative reflex quantitative estimation was performed and level was 2.0 U/g Hb (reference range 4.60–13.5) and that further confirmed the diagnosis.

## Discussion and conclusions

### Diagnosis

He was presented to us with a history of likely exposure to leptospira organisms, and symptoms that are suggestive of leptospirosis [11] which is a common infection in his locality. In the same week he got admitted, we got several cases of leptospirosis including three patients with Weil’s disease. His physical examination findings were also compatible with leptospirosis. Hence it was our first differential diagnosis. A dengue epidemic was also going on in his locality. We do not usually see icterus in dengue, especially within 3 days after the onset of fever although dengue patients also can present with a similar signs and symptoms. The color of his urine sample during our second assessment in day 1 at hospital and his pallor and icterus prompted us to ask for more details and to do a workup for acute hemolysis. That helped us to come to the correct diagnosis and manage his problem. Many clinicians we know give less emphasis to inspection of urine in the present era (compared to two decades ago). However, that made a big impact in the diagnosis of this patient. His presenting symptoms can be attributed to hemolysis in a G6PD deficient patient [10]. Hemolysis induced by leptospirosis (or even by another infection) in this G6PD deficient patient was also a possibility. Acute hemolysis can occur due to leptospirosis per se even in normal people [13]. However, he had no fever after admission and despite of the stopping of ceftriaxone after the first dose, he recovered promptly. That excludes those possibilities. Nonetheless, had he had leptospirosis as well, the diagnosis and management of the patient would have been more difficult. The great majority of Sri Lankan hospitals do not have the facilities to perform serological or PCR tests for leptospirosis making confirmation of the diagnosis very difficult.

#### Acalypha indica induced hemolysis in G6PD deficient person

All reported cases [6–9] and other cases we have seen of *Acalypha indica* induced hemolysis in a G6PD deficient person were subsequent to the use of it as an alternative medicine. Usually they add other medicines also to the herbal broths. But this patient had eaten it as a vegetable in the form of typical Sri Lankan dish “*mellum (mallung)*” (leaves shredded and cooked with grated coconut kernel). *Acalypha indica* grows in many regions of tropical Asia and Africa and America and is used as a medicinal plant as well as a vegetable [5]. Nonetheless all except one report of *Acalypha indica* induced hemolysis in G6PD deficient patients are from Sri Lanka. The only other report we found was from the neighboring Tamil Nadu state of India [9]. That may be mere coincidence or may be because some G6PD deficiency genotypes and G6PD enzyme phenotypes common among Sri Lankans are more susceptible to *Acalypha indica* induced hemolysis. The prevalence G6PD gene variants among Sri Lankans were demonstrated to be different from other Asian populations [4]. That supports the speculation stated before. However, at present tests for G6PD gene variants are unavailable at Sri Lankan hospitals, and even at the national Medical Research Institute-Colombo. Hence, we were unable to determine the G6PD deficiency genotypes of our patient. Further studies are necessary for a clarification.

#### Severe hemolysis

On day 2 his hemoglobin was 5.9 g/dl even after the transfusion of 700 ml packed cells. He had eaten a large portion of the dish to stress to his children the importance of eating vegetables. That may have contributed to severe hemolysis. There is a published study on predictors of severe hemolysis with G6PD deficiency following exposure to oxidants stress [14]. They reported those predictors as the male gender, negative family history, presence of fever and vomiting, a younger age and high Alkaline phosphatase levels. This study was done on a pediatric population [14]. Our patient was an adult but he also had those factors, except the last two.

## Conclusions

Hemolytic crisis following oxidative stresses in G6PD deficient patients can present mimicking leptospirosis. The inspection of urine can give a useful clue towards the correct diagnosis. Consumption of *Acalypha indica* leaves as a vegetable can results in severe hemolytic anemia in some G6PD deficient patients. Screening and awareness campaigns regarding G6PD deficiency in high prevalence areas can reduce the probability of such patients getting a life-threatening hemolytic crisis as in this case. Further studies may reveal whether certain G6PD deficiency genotypes and G6PD enzyme phenotypes common among Sri Lankans are more susceptible to *Acalypha indica* induced hemolysis.

## Abbreviations

G6PD: glucose-6-phosphate dehydrogenase
PCR: polymerase chain reaction
SNP: single-nucleotide polymorphisms
